# Mild folate deficiency induces genetic and epigenetic instability and phenotype changes in prostate cancer cells

**DOI:** 10.1186/1741-7007-8-6

**Published:** 2010-01-21

**Authors:** Gaia Bistulfi, Erika VanDette, Sei-Ichi Matsui, Dominic J Smiraglia

**Affiliations:** 1 Department of Cancer Genetics, Roswell Park Cancer Institute, Elm & Carlton Streets, BLSC L3-314, Buffalo, NY 14263, USA

## Abstract

**Background:**

Folate (vitamin B9) is essential for cellular proliferation as it is involved in the biosynthesis of deoxythymidine monophosphate (dTMP) and s-adenosylmethionine (AdoMet). The link between folate depletion and the genesis and progression of cancers of epithelial origin is of high clinical relevance, but still unclear. We recently demonstrated that sensitivity to low folate availability is affected by the rate of polyamine biosynthesis, which is prominent in prostate cells. We, therefore, hypothesized that prostate cells might be highly susceptible to genetic, epigenetic and phenotypic changes consequent to folate restriction.

**Results:**

We studied the consequences of long-term, mild folate depletion in a model comprised of three syngenic cell lines derived from the transgenic adenoma of the mouse prostate (TRAMP) model, recapitulating different stages of prostate cancer; benign, transformed and metastatic. High-performance liquid chromatography analysis demonstrated that mild folate depletion (100 nM) sufficed to induce imbalance in both the nucleotide and AdoMet pools in all prostate cell lines. Random oligonucleotide-primed synthesis (ROPS) revealed a significant increase in uracil misincorporation and DNA single strand breaks, while spectral karyotype analysis (SKY) identified five novel chromosomal rearrangements in cells grown with mild folate depletion. Using global approaches, we identified an increase in CpG island and histone methylation upon folate depletion despite unchanged levels of total 5-methylcytosine, indicating a broad effect of folate depletion on epigenetic regulation. These genomic changes coincided with phenotype changes in the prostate cells including increased anchorage-independent growth and reduced sensitivity to folate depletion.

**Conclusions:**

This study demonstrates that prostate cells are highly susceptible to genetic and epigenetic changes consequent to mild folate depletion as compared to cells grown with supraphysiological amounts of folate (2 μM) routinely used in tissue culture. In addition, we elucidate for the first time the contribution of these aspects to consequent phenotype changes in epithelial cells. These results provide a strong rationale for studying the effects of folate manipulation on the prostate *in vivo*, where cells might be more sensitive to changes in folate status resulting from folate supplementation or antifolate therapeutic approaches.

## Background

**Figure 1:**
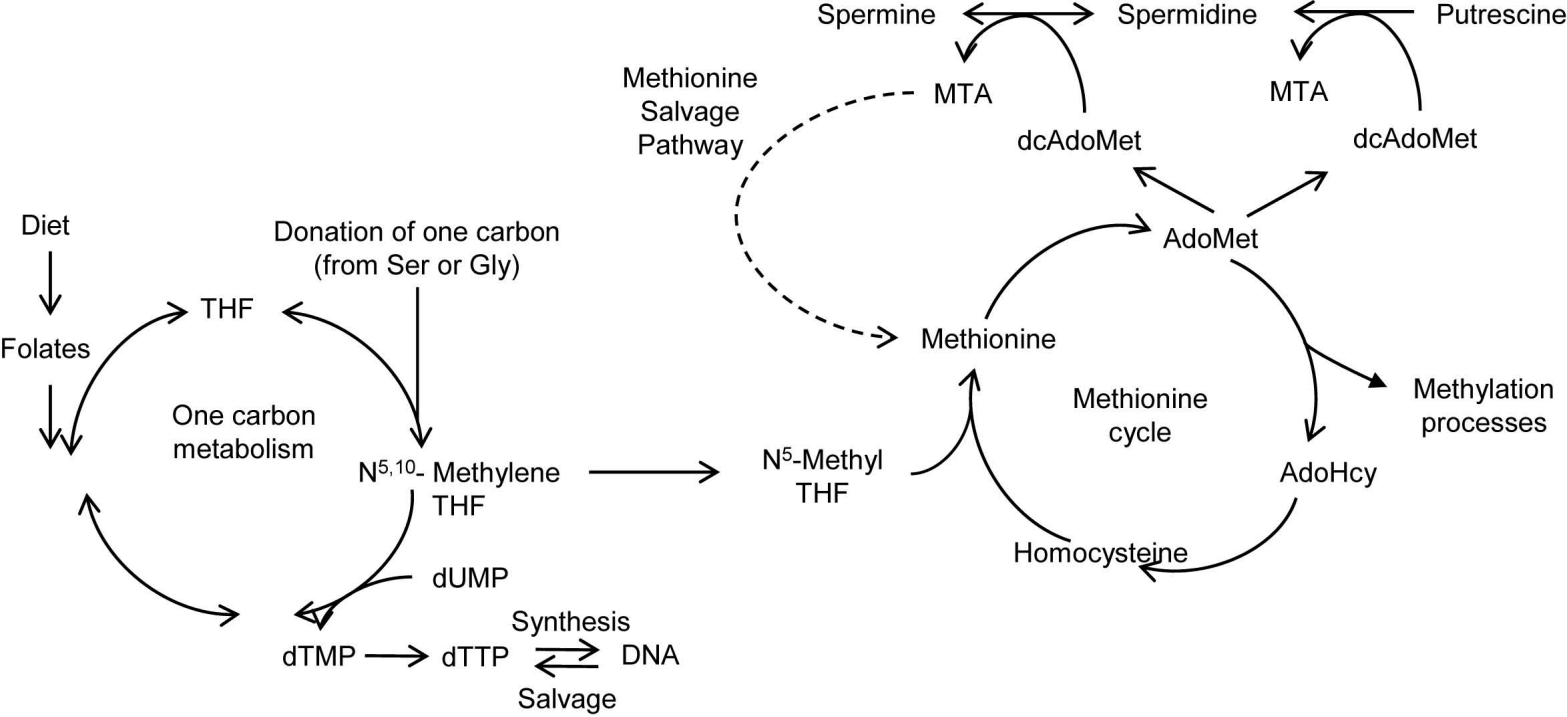
**Folate, one-carbon metabolism, methionine cycle and polyamine biosynthesis overview**. Dietary folate (left) is necessary for *de novo *synthesis of deoxythymidine monophosphate (dTMP) and adenosylmethionine (AdoMet), which are necessary for DNA synthesis and intracellular methylation reactions, respectively. AdoMet is also used for polyamine (spermidine and spermine) biosynthesis (top). dTMP can also be scavenged by DNA degradation as thymidine, while AdoMet can be synthesized independently of folate by recycling methylthioadenosine (MTA) into methionine through the methionine salvage pathway.

Folate (vitamin B9) is an essential nutrient required for the *de novo *synthesis of deoxythymidine monophosphate (dTMP) and s-adenosylmethionine (AdoMet) through one-carbon metabolism and methionine cycle, respectively (Figure 1). dTMP is then converted into the triphosphate form (dTTP), which is required for the synthesis of DNA, while AdoMet is pivotal in a number of metabolic pathways, including the biosynthesis of polyamines (reviewed in [1]), and intracellular methylation reactions that involve DNA, RNA and proteins, including histones (reviewed in [2]).

Dietary manipulation of folate has been referred to as a 'double edge sword' because pharmacological depletion of folate by antifolate drugs prevents cancer cell proliferation but might induce genetic and epigenetic damage and consequent transformation in non-cancerous cells. Conversely, folate supplementation might prevent transformation but sustain the high proliferation rates characteristic of cancer tissue [3]. Epidemiological studies associating dietary intake of folate with the incidence of different cancer types are often conflicting likely reflecting this apparent paradox [3]. Molecular studies, both *in vitro *and *in vivo*, suggest that indeed the effects of folate depletion might depend on the cell type [4] and on the cellular stage of transformation [5]. Strikingly, dietary folate depletion, alone or combined with a low methyl diet, induced tumorigenesis of liver and colon in rodents [6,7].

Based on seminal data from multiple laboratories it was hypothesized that the mechanism of carcinogenesis induced by folate depletion might include both genetic damage consequent to altered availability of dTTP, and epigenetic damage consequent to altered levels of AdoMet [8,9]. Decreased biosynthesis of dTMP, reflected by an increase of dUTP (deoxyuridine triphosphate; Figure 1), leads to uracil misincorporation into the DNA, which is expected to culminate in futile cycles of uracil excision, single strand breaks and possibly chromosomal breakage [8]. Epigenetic damage consequent to folate depletion is a more controversial topic, as altered levels of AdoMet and s-adenosylhomocysteine (AdoHcy; Figure 1) together with changes in the global levels of DNA methylation are not consistently found in different cell types [4,5]. Also, altered CpG island methylation was found in the promoter of p53 and p16 of rodents fed a folate/methyl deficient diet [6,10] raising the possibility that folate depletion might have a global effect on CpG island methylation.

We recently demonstrated that one of the factors affecting cellular sensitivity to folate depletion is the rate of polyamine biosynthesis (spermine and spermidine), which draws on AdoMet pools [11] (Figure 1). Polyamines are ubiquitous molecules essential for cellular life whose intracellular concentration is tightly regulated and maintained in the millimolar range in all cell types [1]. Nonetheless, polyamine biosynthesis is characteristically high in certain tissues including prostate, which, in addition to synthesizing polyamines for epithelial cell replacement, secretes massive amounts of polyamines in the prostatic fluid. Interestingly, a recent clinical trial designed to study the effects of dietary folate supplementation on the prevention of colorectal adenomas reported as the most significant result a five time increase in prostate cancer incidence [12,13]. This finding supports the idea that prostate cells, whose response to folate depletion was never studied before, might be highly dependent on folate in order to sustain their proliferation and, therefore, might be exquisitely sensitive to folate depletion. Thus, we hypothesized that prostate cells might be exquisitely sensitive to genetic, epigenetic and phenotypic changes consequent to folate depletion.

We present a study that, for the first time, simultaneously investigates several aspects characteristic of the molecular, genetic, epigenetic and cellular response to folate depletion in a model comprised of three syngenic prostate cell lines derived from the transgenic adenoma of the mouse prostate (TRAMP) mouse model recapitulating the different stages of prostate cancer - benign, transformed and metastatic - in order to address the possibility that different stages of transformation might alter the response to folate depletion. Indeed, it was previously shown that the effects of mild folate depletion on both the methionine pathway and the methylation status differ among transformed and untransformed human colon cell lines [5]. Our study, using three syngenic but phenotypically distinct cell lines, addresses this possibility.

We report that prostate cells suffer genetic and epigenetic instability along with changes in cell phenotype, even in the presence of levels of folate (100 nM), that allow for the undisturbed growth of other cell types known to be sensitive to more severe folate depletion. Specifically, we demonstrate for the first time that long-term (20 population doublings; PD), mild folate depletion alone is sufficient to induce chromosomal rearrangements and CpG island hypermethylation changes in prostate cells. The analysis of cellular response to mild folate depletion, integrating one-carbon metabolism, methionine cycle and polyamine biosynthesis, with genetic and epigenetic damage, and consequent phenotypical changes in three distinct prostate cell lines, helps to clarify the interplay and contribution of each of these pathways to carcinogenesis consequent to folate depletion. These findings provide a strong rationale for studying the effects of dietary manipulation of folate intake on the prostate *in vivo*.

## Results

### Chronic mild folate depletion affects prostate cells' phenotype

We grew three syngenic, but phenotypically distinct, clonal cell lines; C-2D (benign), C-2G (tumourigenic) and C-2H (metastatic), which were generated from a primary prostate tumour in a TRAMP mouse [14], in folate-restricted (100 nM) medium for 20 PD and refer to these cells as D100, G100, and H100, respectively. In parallel, we grew the cells for 20 PD with the supraphysiological levels of folic acid (2 μM) routinely used in tissue culture and refer to these cells as DCtrl, GCtrl and HCtrl. As expected, D100, G100 and H100 were significantly depleted of folate (*P *< 0.01, data not shown). The intracellular content of folate ranged between 20 and 70 ng/5 million cells for DCtrl, GCtrl and HCtrl but was <10 ng/5 million cells for D100, G100 and H100. We have previously demonstrated that, although colon cancer cell lines grow normally at 100 nM folic acid (FA), prostate cancer cell lines, due to their high polyamine production, slow their growth to varying degrees with this level of FA in the medium [11]. In order to investigate for any permanent phenotypical change that folate depletion might have induced in the three cell lines we put them back into control medium for 2 PD and tested them for their proliferation rates, anchorage independent growth ability and sensitivity to folate depletion.

**Figure 2:**
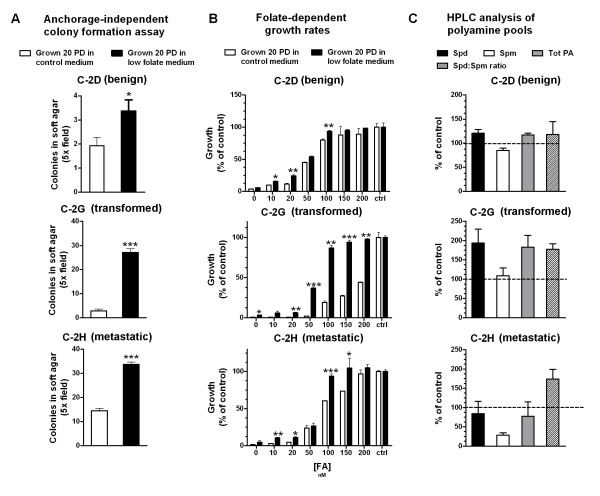
**Chronic mild folate depletion affects prostate cells' phenotype**. (A) Anchorage-independent colonies in soft agar. 10,000 cells well were seeded in semi-solid agar and allowed to grow for 4 weeks. Data presented is the average and SD of six replicates. (B) Folate dependent growth rates. Growth rates (average and standard deviation) are calculated normalizing to growth in control amounts of folate (2 μM) set as 100%. (C) High-performance liquid chromatography analysis of polyamine pools. Results are the average and standard deviation of two biologically independent samples. Values are normalized to those obtained in GCtrl cells, set as 100%. * = *P *< 0.05, ** = *P *< 0.01, *** = *P *< 0.001.

G100, but not D100 and H100, slightly increased their proliferation rate compared to their respective controls, as they decreased their population doubling time from 15.8 h to 14 h (data not shown). All three cell lines grown for 20 PD in low folate medium significantly increased their ability to form anchorage independent colonies in soft agar over their respective controls (Figure 2A). DCtrl, which is derived from the untransformed cell line C-2D, could only form sporadic colonies in soft agar (Figure 2A, top). Growth in low folate for 20 PD slightly, but significantly (*P *< 0.05), increased the number of D100 cells capable of anchorage independent growth in soft agar (Figure 2A, top). Folate depletion had a more dramatic effect on 2G and 2H cells, where the number of colonies per field increased from two for GCtrl to 27 for G100 and from 14 for HCtrl to 33 for H100 (Figure 2A, bottom; *P *≤ 0.001 each).

Interestingly, all cells grown with restricted amounts of folate also significantly (*P *< 0.001 by two-way ANOVA for all three cell lines) decreased their sensitivity to folate depletion as we established by assessing folate-dependent growth curves on all six cell lines (Figure 2B). All three cell lines that had been grown for 20PD in low folate conditions demonstrated nearly 100% growth at 100 nM FA (Figure 2B, black bars), while the DCtrl, GCtrl, and HCtrl versions of the cell lines only grow to 80%, 19% and 60% (Figure 2B, white bars). As expected, the total polyamine production was not significantly affected by growth in low folate (Figure 2C), confirming that maintaining polyamine pools might be a priority in prostate cells, thereby further increasing the stress on AdoMet pools in a folate depleted state (Figure 1). Nonetheless, we found that the spermidine to spermine ratio (spd:spm ratio), which correlates with cancer cells' aggressiveness and proliferation rates in several cell types *in vivo *[15] , was increased in G100 and H100 as compared to control cells (177% and 174%, respectively; Figure 2C).

### Chronic mild folate depletion induces DNA damage in prostate cells *in vitro*

**Figure 3:**
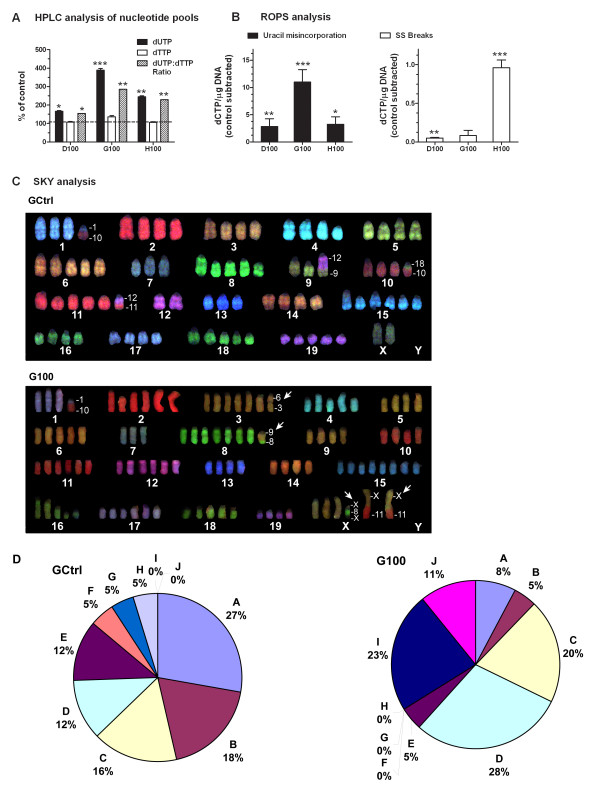
**Mild folate depletion induces genetic damage in prostate cells**. (A) High-performance liquid chromatography analysis of nucleotide pools. Results are the average and standard deviation of three independent injections. Values are relative to cells grown in control medium. (B) Single strand breaks generated by treatment with uracil-DNA-glycosidase (UDG) and Exonuclease III are quantified by random oligonucleotide-primed synthesis performed with the Klenow polymerase in the presence of P^32^-deoxcytidine triphosphate (dCTP; left). The values of dCTP incorporation for all cell lines are the average of five replicates after subtraction of no enzyme treatment control background and presented as an increase over cells grown in control medium. (C) Spectral karyotype analysis reveals that mild folate depletion induced *de novo *chromosomal aberrations in G100. A representative metaphase from GCtrl (top), which exhibits only marker chromosomes common to both GCtrl and G100 (Table 1) and a representative metaphase from G100 (bottom) which harbours the common *t*(1;10) translocation along with four marker chromosomes, indicated by arrows, found frequently in G100, but never in GCtrl (Table 1). (D) Distribution of metaphase cells with frequent patterns of marker chromosomes shared between GCtrl and G100 indicates lack of clonal selection in G100. Ten different patterns (A-J) of the five marker chromosomes (a-e; Table 1) were found in two or more metaphase cells. G100 retains significant heterogeneity of patterns of marker chromosomes including two novel patterns (I and J). Pattern A represents marker chromosomes a, b, c and d; pattern B = marker a; pattern C = markers a and e; pattern D = marker b; pattern E = no marker chromosomes; pattern F = markers a, b and c; pattern G = markers b, c and d; pattern H = markers b and c; pattern I = a, b, c, d and e; pattern J = marker e. * = *P *< 0.05, ** = *P *< 0.01, *** = *P *< 0.001.

Analysis of nucleotide pools by high pressure liquid chromatography (HPLC) identified significantly increased intracellular dUTP levels in D100, G100 and H100, up to 166%, 388% and 244% of DCtrl, GCtrl and HCtrl, respectively (Figure 3A). Surprisingly, dTTP was not decreased in any of the three cell lines upon mild folate depletion. dTTP was, in fact, unchanged in both D100 and H100 and increased to 136% in G100 as compared to GCtrl (Figure 3A, middle). Nonetheless, due to the dUTP increase, the dUTP:dTTP ratio was significantly increased in all three cell lines to 155%, 285% and 229% of their respective controls (Figure 3A). In order to determine if the increased dUTP:dTTP ratio led to increased uracil misincorporation into the DNA we analysed the cells' DNA by random oligo-primed synthesis (ROPS) [16,17] and detected significant uracil misincorporation in all three cell lines (*P *< 0.01, *P *< 0.001, and *P *< 0.05 for D100, G100, and H100, respectively; Figure 3B, left) and increased amount of DNA single strand breaks in D100 and H100 (*P *< 0.01 and *P *< 0.001, respectively; Figure 3B, right).

In order to investigate if these static measures of increased uracil misincorporation and single strand DNA breaks were associated with accumulation of chromosomal rearrangements we analysed G100 and GCtrl cells by Spectral Karyotype Analysis (SKY) [18]. We found that G100 and GCtrl shared five marker chromosomes in common, which were seen in greater than 15% of metaphases studied (*n *= 52 for GCtrl and *n *= 76 for G100). As shown in Table 1, G100 cells exhibited five additional marker chromosomes seen in 24% - 37% of cells, which were never seen in any of 52 metaphase cells in GCtrl. A representative metaphase cell from each cell line is shown in Figure 3C demonstrating four of the common marker chromosomes in GCtrl and four of the new marker chromosomes in the G100 cell line (Figure 3C, arrows). Looking at only the marker chromosomes present in both cell lines (a-e, Table 1), we observed eight different patterns of these marker chromosomes in two or more cells in GCtrl and seven different patterns in G100, indicating significant cellular heterogeneity is retained in the G100 cells. Figure 3D shows the frequency distribution of the various patterns (A-J) of these five marker chromosomes in both cell lines. Two novel patterns, I and J, were seen in the G100 cell line, while three relatively rare patterns (H, G, and F) from the GCtrl line were not observed in G100. These data do not support the hypothesis that clonal selection occurred in the G100 cell line. Furthermore, once the five novel marker chromosomes seen in G100 are taken into consideration, it is clear that there is increased, rather than decreased heterogeneity in the G100 cell line.

**Table 1:** Frequency of marker chromosomes detected by spectral karyotype analysis in >15% of metaphases analysed in GCtrl or G100.

Cytogenetic event	Frequency in Gctrl (*n *= 52 metaphase cells)	Frequency in G100 (*n *= 76 metaphase cells)
a* t(11;12)	63%	55%

**b **t(1;10)	54%	58%

**c **t(10;18)	40%	34%

**d **t(6;19)	37%	33%

**e **Y	19%	51%

**f **rcpt(4;9)	0%	**37%**

**g **t(8;9)	0%	**29%**

**h **ins(X;8;X)	0%	**26%**

**i **t(X;11)	0%	**26%**

**j **t(3;6)	0%	**24%**

*Lowercase letters in bold indicate the name of the marker chromosome used to describe the patterns of marker chromosomes in Figure 3

### Chronic mild folate depletion affects AdoMet pools

**Figure 4:**
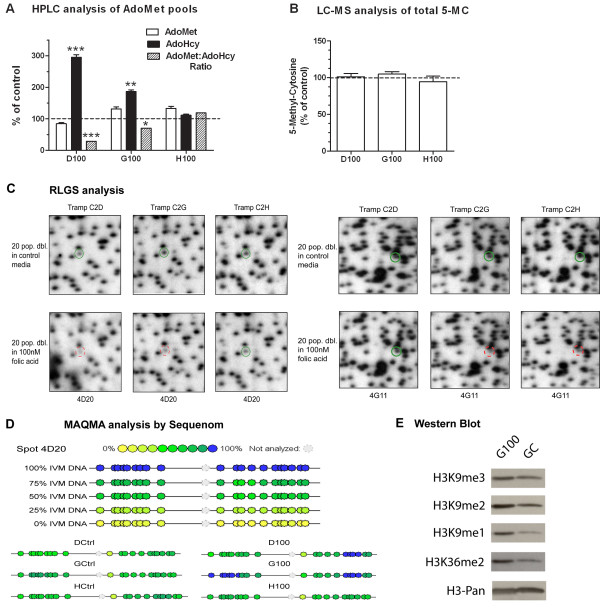
**Mild folate depletion induces epigenetic damage in prostate cells**. (A) High-performance liquid chromatography analysis of adenosylmethionine/adenosylhomocysteine pools. Results are the average and standard deviation of three independent injections and are presented relative to cells grown in control medium (100%). * = *P *< 0.05, ** = *P *< 0.01, *** = *P *< 0.001. (B) Analysis of total levels of 5-methylcytosine by liquid chromatography-mass spectrometry. Results are the average and standard deviation of two independent injections. Values are relative to cells grown in control medium. (C) Sections of restriction landmark genomic scanning (RLGS) gels including the spot 4D20, which shows decreased intensity in both D100 and G100, and the spot 4G11, which shows decreased intensity in both G100 and H100. (D) Mass array quantitative methylation analysis confirmed the methylation status of CpG identified by RLGS and associated to known loci. Peripheral blood lymphocyte DNA was used as a 0% methylation control and an *in vitro *methylated aliquot of the same DNA as a 100% methylated control. These DNAs were mixed at appropriate ratios to generate 75%, 50% and 25% methylated controls. The figure shows 4D20 as an example. (E) Western Blot analysis of histone acid lysates run on sodium dodecyl sulphate-page gel revealed an increase of global H3K9 methylation (17 Kda) and H3K36 di-methylation (17 Kda) upon mild folate depletion.

We measured AdoMet and AdoHcy by HPLC in the three cell lines grown with or without mild folate depletion, as shown in Figure 4A. AdoMet levels in D100 were reduced to 84% of those found in DCtrl cells. Conversely, AdoMet was increased upon folate depletion in G100 and H100, where it was 131% and 132% of GCtrl and HCtrl, respectively. As a consequence of folate depletion, AdoHcy was increased in all cell lines as it reached 295%, 186% and 111% of levels detected in DCtrl, GCtrl, and HCtrl, respectively (Figure 4A). The AdoMet:AdoHcy ratio upon mild folate depletion was consequently decreased in D100 and G100 (28% and 70%, of DCtrl and GCtrl, respectively) and increased in H100 (118%; Figure 4A). Regardless of the changes observed in the AdoMet:AdoHcy ratio, we found no significant change in the total levels of 5-methylcytosine in the genome of D100, G100 and H100 compared to their respective controls as measured by liquid chromatography paired to mass spectrometry (LC-MS; Figure 4B) [19].

### Increased CpG island methylation and histone hypermethylation

Folate depletion has been associated with *de novo *CpG methylation of candidate genes in colon and liver *in vivo *[6,10,20]. In order to determine if mild folate depletion induced global CpG island hypermethylation in the prostate cell lines, we measured CpG island methylation by restriction landmark genomic scanning (RLGS); a global approach that allows for the simultaneous screening of the methylation status of over 1200 distinct CpG islands in the genome. Each spot is named according to a coordinate naming system as previously described [21] and the sequence represented by approximately half of the spots has been identified [22]. Comparing the three cell lines grown in 100 nM FA to the same cells grown in control medium we identified 14 hypermethylated loci, as defined by RLGS spot loss [21,23]. As shown in Table 2, three RLGS spot methylation events were found in two of the three cell lines. For eight of the RLGS loci, the sequences represented by the spot are known (Table 2).

**Table 2:** Restriction landmark genomic scanning (RLGS) methylation events detected in the three cell lines after growth in 100 nM folic acid for 20 population doublings.

Spot name	D100	G100	H100	Gene name
**1E09**	--	--	MET	Igf2bp3

**2B45**	--	MET	MET	Prodh

**2C01**	--	MET	--	Mapk4

**2D25**	--	MET	--	Cnnm1

**2D47**	--	MET	--	AK147279

**3C21**	--	MET	--	Nrxn2

**4D20**	MET*	MET	--	Argef7

**4G11**	--	MET	MET	Gm967

**1E08**	--	MET	--	NA

**2D15**	--	MET	--	NA

**2F43**	--	MET	--	NA

**2G58**	--	MET	--	NA

**5G29**	--	MET	--	NA

**6G44**	--	MET	--	NA

*MET indicates methylation of the NotI site represented by the RLGS spot.

Similar to what was observed for changes in cell phenotype and uracil misincorporation, G100 had the most dramatic change in methylation pattern with 13 hypermethylated loci, followed by H100 with three and D100 with one. Figure 4C shows representative regions of the RLGS gels for each cell line demonstrating methylation of two RLGS spots (4D20 and 4G11). Note that spot 4D20, corresponding to a CpG island 5' to the Argef7 gene, shows greatly reduced intensity (increased methylation) in both D100 and G100 cells compared to control (Figure 4C, left), while the spot 4G11, corresponding to a CpG island 5' to the Gm967 gene, displayed decreased intensity in both G100 and H100 cells (Figure 4C, right).

In order to confirm that the decrease in spot intensity identified by RLGS corresponded to an actual increase of DNA methylation in the corresponding CpG islands, we utilized the Sequenom platform for quantitative bisulfite sequencing of the eight identified RLGS spots. Mass array quantitative methylation analysis (MAQMA) confirmed all the RLGS spot methylation events listed in Table 2. Figure 4D shows the hypermethylation of 4D20 in D100 and G100, but not H100, compared to the control cell lines.

In order to determine if mild folate depletion induced any change in the global levels of histone methylation in prostate cells like it was reported in other cell types [24], we assessed global methylation levels at key residues of histone H3 (H3) in the G100 and GCtrl cell lines. Using Western blot analysis of histones we measured H3 lysine 9 (K9) methylation, which is well characterized and known to associate with a repressive transcriptional status and CpG island hypermethylation. We found that mono-, di- and tri-methylation were all increased at H3K9 in G100 cells (Figure 4E). In order to understand if the increase in histone methylation was specific to transcriptionally repressive histone modifications we analysed the levels of histone H3 lysine 36 di-methylation (H3K36me2), as this modification is known to associate with the body of transcriptionally active genes. We found that H3K36me2 was also increased in cells grown with mild folate depletion. This demonstrates that the increase in histone methylation induced by mild folate depletion is not specific to repressive modifications and is probably not directly related to the relatively low level of increased CpG island hypermethylation.

## Discussion

In this study we hypothesized that prostate cells might be exquisitely sensitive to genetic, epigenetic and phenotypic changes consequent to folate depletion and investigated the potential role that different stages of transformation might play in affecting the response to folate depletion. This topic is clinically relevant as antifolate drugs are currently used in the clinic for the treatment of certain types of cancer as well as infections from opportunistic agents [25]. We found that even a mild folate depletion (100 nM) was sufficient to induce dramatic genetic and epigenetic damage along with changes in prostate cell behaviour compared with cells grown with the supraphysiological levels of folate routinely used in tissue culture (2 μM). One hundred nanomolar of folate is about five times the amount that negatively impacts cell types such as colon, already known to be sensitive to folate depletion [5,11,26,27]. The prostate cells' exquisite sensitivity to folate depletion is consistent with their characteristically high polyamine biosynthesis, which results in extra demand on the methionine cycle and one-carbon metabolism in order to sustain AdoMet pools [11].

The levels of polyamine biosynthesis might be one of the factors behind the different response to folate depletion in cells at different stages of transformation. Polyamine levels are significantly increased during the process of transformation in order to sustain cell hyperproliferation [1,28]. Throughout our study, the untransformed cell line C-2D suffered less dramatic changes both at the molecular and phenotypical levels, consistent with C-2D having the lowest polyamine levels among the three cell lines [11].

Another mechanism that might differentiate the cells' response to folate depletion could be their ability to efficiently repair DNA. Cells at more advanced stages of transformation are more likely to have impaired DNA repair machinery and might accumulate mutations at a higher rate. Consistent with this hypothesis, although for all three cell lines the levels of uracil misincorporation followed closely the increase in the dUTP:dTTP ratio (Figure 3A and 3B) - which in turn was more dramatic in cells with higher polyamine biosynthesis - the levels of DNA damage measured as DNA single strand breaks did not (Figure 3B and 3C). This suggests that even if the dUTP:dTTP ratio and the levels of uracil misincorporation were strictly dependent on the inherent folate sensitivity of the cell lines and, therefore, polyamine biosynthesis, the ultimate amount of DNA damage might also depend on the cells' ability to repair the DNA efficiently. Therefore, it is not surprising that we found the highest amount of DNA single strand breaks in the metastatic cell line C-2H (Figure 3C).

In addition we found that folate depletion by itself, even at 100 nM folic acid, can induce major heritable chromosomal rearrangements in prostate cells. Whilst it has long been known that low folate levels can induce fragile sites in both human and mouse chromosomes [29-31], whether or not expression of the fragile sites is associated with chromosomal rearrangements is unclear. Spectral Karyotype Analysis (SKY) of the C-2G cells grown with or without mild folate deficiency, identified several novel chromosomal rearrangements upon mild folate depletion (Table 1 and Figure 3), which are probably linked to the altered nucleotide pools, uracil misincorporation and single strand DNA breaks we observed in the same cells. We found that a number of the breakpoints for chromosomal translocations mapped in the vicinity of known fragile sites, including a break at 6qC1 (marker chromosome **i**; Table 1) corresponding to Fra6C1 in mouse and the human FRA4F [32].

Importantly, these novel chromosomal rearrangements were never seen in the parental cell line and, in fact, resulted in increased chromosomal heterogeneity (Figure 3D), therefore discounting the possibility of clonal selection of pre-existing changes. This is also important when considering the decreased sensitivity to folate depletion that the cells acquired after growth with limiting amounts of folate (Figure 2B). We suggest that cells might have adapted to growth in low folate through up regulation of certain key pathways in order to sustain their proliferation. In support of this hypothesis, when we analysed molecule pools by HPLC, we observed that despite imbalance in both the dUTP:dTTP ratio and AdoMet:AdoHcy ratio, the overall levels of key molecules, namely dTTP and AdoMet were maintained (Figures 3A and 4A).

An intriguing possibility is that upregulation of the thymidine salvage pathway and the methionine salvage pathway might occur in these cells upon mild folate depletion (Figure 1). The thymidine salvage pathway scavenges thymidine from degraded nucleic acids and would, therefore, help in sustaining dTTP levels independent of uracil conversion (Figure 1). Such a scenario would be expected to result in an accumulation of dUMP due to a lack of methylation to dTMP, while at the same time result in the sustainment of dTTP pools due to salvage. Similarly, *de novo *synthesis of AdoMet is mainly accomplished by the recycling of AdoHyc to homocysteine, followed by N^5^-methyl tetrahydrofolate dependent synthesis of methionine, and is, therefore, dependent on one-carbon metabolism. However, the methionine salvage pathway, which recycles methylthioadenosine out of polyamine biosynthesis into methionine (Figure 1) could contribute to AdoMet biosynthesis independently of folate and result in sustained AdoMet levels without utilization of AdoHcy, which would accumulate as a byproduct of DNA, RNA and protein methyltransferase reactions. This pathway has been described mainly in liver, but its rate limiting enzyme (methylthioadenosine phosphorylase) is ubiquitous, was originally purified from rat prostate [33,34] and is highly expressed in all three cell lines (data not shown) used in this study.

Another novel contribution of this work is the demonstration that mild folate depletion induced global CpG island hypermethylation in all three prostate cell lines. Some of the methylation events were common among cell lines, suggesting that CpG island hypermethylation under conditions of folate depletion might not be occurring randomly. Interestingly, in all the CpG island hypermethylation cases we observed, some methylation was also present in the control cell lines which suggests that mild folate depletion did not induce completely *de novo *methylation at these promoters but, rather, increased hypermethylation at already susceptible loci. Conversely, despite the decreased AdoMet:AdoHcy ratio in D100 and G100 cells, we could not detect global DNA hypomethylation as assessed by LC-MS (Figure 4B). This result was somewhat surprising but does not preclude the possibility that mild folate depletion induced hypomethylation at satellite repeats, as was previously reported in colon cell lines *in vitro *[5]. Consistent with previous findings in the rat liver [24,35], we also found that, in prostate cells, mild folate depletion induced global histone hypermethylation regardless of whether the specific marks were associated with increased or decreased transcription (Figure 4E). All these events, genetic and epigenetic, probably collaborate, resulting in the altered phenotype of the cells that we observed (Figure 2).

## Conclusions

Prostate cancer is the second leading cancer causing mortality in men in the USA [36]. Our data show that prostate cells are exquisitely sensitive to folate depletion, with major genetic and epigenetic consequences resulting in altered cellular phenotypes. Insufficient dietary folate intake or antifolate therapies, which are used for the treatment of certain type of cancers as well as infections by opportunistic agents [25], might increase the incidence of prostate cancer due to the potentially negative effects on the genome. Folate supplementation on the other hand, although beneficial to untransformed cells, might, in fact, accelerate the growth of existing, dormant prostate cancer cells. This hypothesis is supported by the results of a recent clinical trial designed to study the effects of folate supplementation on colon polyp prevention, which resulted in a significant and unexpected increase in the incidence of prostate cancer [12,13]. Conversely, the heightened sensitivity of prostate cancer cells to folate deficiency suggests that therapeutic use of antifolates might be particularly effective in prostate cancer patients.

## Methods

### Cell culture

The three syngenic, but phenotypically distinct, clonal cell lines C-2D (benign), C-2G (tumourigenic) and C-2H (metastatic) were generated from a primary prostate tumour in a TRAMP mouse [14] and were phenotypically defined by their ability to form tumours and metastasis *in vivo *[14]. All cell lines were grown in folate-free RPMI-1640 (Invitrogen, CA, USA) plus 10% dialyzed fetal bovine serum (Invitrogen) supplemented with 10^-8 ^dihydrotestosterone and either 100 nM FA (Sigma-Aldrich, MO, USA; low folate) or 2 μM FA (control) for 20 PDs. Medium was changed every 2 PD.

### Anchorage-independent colony formation assay in soft agar

Anchorage-independent colony formation assay in soft agar was performed as previously described [37]. Briefly, 10,000 cell/well were seeded in semi-solid agar mixed with medium in six replicates in six well plates. Agar was covered with medium which was replaced once a week. After 4 weeks, five fields per well were assessed microscopically for the presence of anchorage-independent colonies > 50 μM. The experiment was repeated twice.

### Folate-dependent growth rates

We seeded 3000 cells/well in six well plates in duplicate with the specified amounts of FA. We carried out counts with Trypan blue after 9 days of culture. For each cell line we set growth in control medium (2 μM FA) as 100% of growth and calculated the relative growth rates in folate restricted media.

### Analysis of intracellular folates by *L. Casei*

Intracellular quantification of folate pools was carried out using a microbiological assay like previously described [11,38].

### HPLC analyses

HPLC analyses were carried out as previously described [11,39,40] at the Biopolymer Facility, at Roswell Park Cancer Institute (NY, USA).

### ROPS

ROPS was carried out as previously described [16,17] in order to measure uracil misincorporation, apyrimidinic sites and DNA single strand breaks. We analysed at least five independent replicates per sample (D100, DC, G100, GC, H100, HC). Uracil misincorporation was normalized, as recommended, by subtracting the background due to pre-existing single strand breaks and apyrimidinic sites. The so-obtained data expressed as deoxcytidine (dCTP) incorporated per microgram of DNA per cell line grown in mild folate depletion were expressed as a percentage of the average value obtained for the same cells grown in control medium. Statistical analysis of ROPS experiments was carried out on the actual dCTP/μg values.

### SKY analysis

SKY was done essentially as described [18]. Briefly, fluorescence colour images of chromosomes developed by Rhodamine, Texas Red, Cy5, FITC and Cy5.5 were captured under a Nikon microscope, equipped with a spectral cube and Interferometer module. SKY View software (version 1.62), was used to sort numerical changes and structural alterations of chromosomes, including simple balanced translocations, unbalanced (or nonreciprocal) translocations, deletions and duplications. At least 50 metaphases were analysed per sample.

### Total 5-methylcythosine analysis

We performed total 5-methylcytosine quantification by hydrolyzing the DNA and performing a separation of nucleotides by LC-MS as previously described [19].

### RLGS

The protocol for extraction of genomic DNA from cells was previously described by Smiraglia *et al*. [41,42]. The published protocol of Dai *et al*. [43] was followed for RLGS gels. RLGS spots of interest were cloned as previously described [22,41,44,45]. The resulting highly reproducible pattern is comprised of over 1200 spots representing as many CpGs distributed genome-wide. Decreased spot intensity on the RLGS gel corresponds to an increase in methylation at the corresponding CpG [23,46].

### MAQMA by Sequenom

Briefly, the DNA was bisulphite treated (EZ DNA methylation kit, Zymo Research, CA, USA) and polymerase chain reaction amplified (HotStarTaq DNA Polymerase, Qiagen, CA, USA) with bisulphite sequencing primers flanking the CpGs of interest at loci identified by RLGS. MAQMA was performed using the MassARRAY Compact system developed by the Sequenome Company (CA, USA) as previously described by Ehrich *et al*. [47]. This system utilizes MS for the detection and quantitative analysis of DNA methylation. This approach has been shown to a be a highly accurate and reproducible way to quantitated DNA methylation [48].

### Western Blot analyses

In order to analyse histone modifications we separated 30 μg of histone acid lysates per sample on a 15% polyacrylamide gel, transferred the proteins onto a polyvinylidene fluoride membrane and immunoblotted with antibodies specific to H3K9me1, H3K9me2, H3K9me3, H3H36me2 and the total level of H3 as a loading control. Detection was performed with horse radish peroxidase-conjugated secondary antibodies and enhanced chemiluminescence detection on film (Pierce, IL, USA). All antibodies were purchased from Millipore (MA, USA). All cells were harvested at 75% confluence.

### Statistical analysis

Statistical significance throughout the paper was calculated with the standard student's t-test (unpaired, two-tailed) unless stated otherwise.

## Abbreviations

AdoHcy: adenosylhomocysteine; AdoMet: adenosylmethionine; dCTP: deoxycytidine triphosphate; dTMP: deoxythymidine monophophate; dTTP: deoxythymidine triphosphate; dUTP: deoxyuridine triphosphate; HPLC: high-performance liquid chromatography; LC: liquid chromatography; MAQMA: mass array quantitative methylation analysis; MS: mass spectrometry; PD: population doublings; RLGS: restriction landmark genomic scanning; ROPS: random oligonucleotide-primed synthesis; SKY: spectral karyotype analysis; TRAMP: transgenic adenoma of the mouse prostate.

## Authors' contributions

GB made substantial contributions to the conception and design of the study, carried out all the experimental work, unless stated otherwise, and wrote the paper draft. EVD contributed to the western blot analysis. SM carried out the SKY analysis. DJS made substantial contributions to the conception and design of the study, carried out the RLGS analysis and contributed in drafting the manuscript. All authors read and approved the final manuscript.
